# The interventionist mindset: The ten eyes rule in cath lab

**DOI:** 10.1016/j.amsu.2020.11.070

**Published:** 2020-11-28

**Authors:** Ameen Mosa Mohammad

**Affiliations:** Duhok Heart Center/ College of Medicine, University of Duhok, 9 Azadi Hospital RRoad, Azadi, Duhok, 1014AM, Kurdistan, Iraq

**Keywords:** Coronary intervention, Percutaneous coronary intervention, Cath lab leader

## Abstract

This article addresses the skillfulness role of the interventionist in the Cath lab. It argues that the interventionist plays a crucial role and should possess certain mental-manual dexterity and hand-eye coordination skills. The article suggests a series of measures that collectively determine the successful role of the interventionist in the Cath lab. This is of utmost importance given the sensitive nature of the cardiovascular procedures, the potential costs of its failure for the patient, and the key action played by the interventionist in determining the failure or success of the procedure.

The cardiovascular interventionist, particularly during coronary intervention, should necessarily have an eagle eye in the cath lab. Coronary intervention is a highly sensitive procedure given that an interventionist can either succeed or fail in performing the required task. Failure is even more costly due to the negative implications for the patient in question [1].

Training in the field contributes to the safety of the interventionist as well as the patient. However, essential background knowledge covering all stages of the pre-operative, operative and post-operative stages of the procedure is required for a successful outcome. For an interventionist in the cath lab, the following ten elements need to be given special attention:

1. Complete familiarity with the cath lab is extremely important for the interventionist before proceeding towards the procedure. This includes the equipment in the lab. This is important for performing a smooth and easy procedure and dealing with alternative plans if necessary [1,2].
2. Sheath: starting with a start the interventionist should avoid needlesticks and monitor the access sheath for any inadvertently incidence of hematoma, withdrawal, and kinking in order to end the procedure either with minimal access complications [3].
3 The manifold system, including the contrast and pressure monitoring: the interventionist should not depend on the experience of thesecond operator if they have one; the interventionist should be watching all aspects of the system. Many shortcomings related with the cath lab procedure rely on the entire system and its functionality [3,4].
4 Haemodynamic monitoring: is vital to assessing the clinical condition of the patient throughout the procedure. Not paying necessary attention to the haemodynamics might lead to preventable complications or fatalities during the intervention. Distraction can happen, particularly when it comes to complications and difficult procedures, but it must be avoided [5].
5 The guide: is the interventionist's tunnel of light towards the target. The interventionist should keep an eye on the guide at all times. One should not miss seeing the tip of the guide at any point during the whole procedure [6].
6 The coronary lesion: can be friendly or not. This behavior partially relies upon the interventionist's eye and approach. An interventionalist must collect as much information as possible on characteristics of the lesion [6,7].
7 The coronary wire: the complaint weapon in your hand. The interventionist should be select the right wire and manipulate it well during through the artery with gentle negotiation-based crossing the lesion. The interventionist should also keep an eye on the end of the tip in the distal portion of the artery to avoid unbridled phenomenon and the associated risk of complications, particularly perforation [[6], [7], [8]].
8 The deliverable interventional tools such as balloons and stents: The interventionist should play his or her role in making the best decision when choosing a tool. The interventionist should wisely select the most appropriate tool for the procedure at hand and should monitor the movement (dynamics) of such tools throughout the procedures [7,8].
9 Self-care and temper: The interventionist should remain patient and afford difficult times. He or she should play the leading role in the cath lab. The interventionist's protection from unnecessary hazards in the lab is also important [2,9].
10 The above-mentioned points are not off the table at any moment and stages of the procedure. The patient remains the top priority and should be closely monitored for his safety and response during the procedure and after it. His or her wellbeing is the ultimate objective. All the necessary cardiac and non-cardiac health data of the indexed patient should be clear to the operator in order to take the necessary measures if needed [2,10].

## Provenance and peer-review

Not commissioned, internally reviewed.

## Ethical approval

Due approval from the ethics committee was obtained.

## Source of funding

No funding to declare.

## Author contribution

AMM: designing the study, drafting and conducting the version of the manuscript.

## Declaration of competing interest

None.
